# A process mining approach to assessing diagnostic pathways in a memory clinic

**DOI:** 10.1002/alz.086783

**Published:** 2025-01-09

**Authors:** Cristina Festari, Claudio Singh Solorzano, Stefania Orini, Simona Buscarnera, Francesco Rossato, Michela Pievani, Matteo Cotta Ramusino, FEDERICO MASSA, Roberto Gatta, Giovanni B. Frisoni

**Affiliations:** ^1^ IRCCS Istituto Centro San Giovanni Di Dio Fatebenefratelli, Brescia Italy; ^2^ Università degli Studi di Brescia, Brescia Italy; ^3^ Sapienza University of Rome, Rome Italy; ^4^ University of Padova, Padua Italy; ^5^ IRCCS Mondino Foundation, Pavia Italy; ^6^ University of Genova, Genova Italy; ^7^ IRCCS Ospedale Policlinico San Martino, Genova Italy; ^8^ Geneva University Hospitals, Geneva Switzerland; ^9^ University of Geneva, Geneva Switzerland

## Abstract

**Background:**

Several clinical guidelines and recommendations have been developed to improve the diagnostic process of patients with dementia, with the aim of optimising patient care and fostering a cost‐effective use of resources. However, patient characteristics and organizational factors in clinical practice can lead physicians to deviate from recommendations. In this context, process mining (PM) is a powerful technique for evaluating the compliance of actual to ideal diagnostic pathways, but has never been used in the cognitive field. This study aims to pilot the use of PM to study the diagnostic workup of patients with cognitive complains in an academic memory clinic.

**Method:**

We retrospectively reviewed medical charts of 539 consecutive new patients referred to the University Hospitals of Geneva (Switzerland) between July 2021 and December 2022. The collected information includes socio‐demographic data, number and dates of clinical visits and biomarker exams (e.g., FDG‐, amyloid‐, tau‐PET, cerebrospinal fluid ‐ CSF). The pMineR package, a R library for PM, was used for data inspection and process discovery (e.g., First Order Markov Model ‐ FOMM, Careflow Miner ‐ CFM) to extract features of most frequent diagnostic pathway according to clinical stage.

**Result:**

Patients were 96 subjective cognitive decline (SCD; MMSE: 28.5±1.6), 308 Mild Cognitive Impairment (MCI; MMSE: 26.5±2.7), and 135 Mild Dementia (DEM; MMSE: 22.7±3.2). FOMM showed that all SCD patients received a syndromic diagnosis based on a detailed neuropsychological evaluation and a morphological imaging. CSF was the first‐line biomarker to ascertain the aetiology of cognitive impairment, used in 90 MCI (30%) and 32 DEM (24%). A second‐line biomarker (e.g., amyloid‐, FDG‐, Tau‐PET) was required in 23% of MCI and 16% of DEM. Differential PM with CFM revealed that DEM was more commonly referred to the first clinical assessment with morphological imaging than MCI (DEM:68/135vs.MCI:123/308, ratio:1.8; p = 0.047).

**Conclusion:**

These preliminary results demonstrate that the PM approach is appropriate for describing the diagnostic workup in memory clinics. Using the same approach and study design, a further project will evaluate the diagnostic pathways in 6 European academic memory clinics and contrast them to recommendations recently issued from a large European consensus effort (Frisoni, LancetNeurol 2024).

